# Tricuspid valve repair for infective endocarditis

**DOI:** 10.1093/icvts/ivae084

**Published:** 2024-04-30

**Authors:** Veronica Lorenz, Stefano Mastrobuoni, Gaby Aphram, Matteo Pettinari, Laurent de Kerchove, Gebrine El Khoury

**Affiliations:** Department of Cardiothoracic and Vascular Surgery, Université Catholique de Louvain, Cliniques Universitaires Saint-Luc, Brussels, Belgium; Department of Cardiothoracic and Vascular Surgery, Université Catholique de Louvain, Cliniques Universitaires Saint-Luc, Brussels, Belgium; Department of Cardiothoracic and Vascular Surgery, Université Catholique de Louvain, Cliniques Universitaires Saint-Luc, Brussels, Belgium; Department of Cardiothoracic and Vascular Surgery, Université Catholique de Louvain, Cliniques Universitaires Saint-Luc, Brussels, Belgium; Department of Cardiothoracic and Vascular Surgery, Université Catholique de Louvain, Cliniques Universitaires Saint-Luc, Brussels, Belgium; Department of Cardiothoracic and Vascular Surgery, Université Catholique de Louvain, Cliniques Universitaires Saint-Luc, Brussels, Belgium

**Keywords:** Endocarditis, Infective endocarditis, Tricuspid valve, Valve repair, Homograft

## Abstract

**OBJECTIVES:**

The progressive increase in the use of implantable electronic devices, vascular access for dialysis and the increased life expectancy of patients with congenital heart diseases has led in recent years to a considerable number of right-side infective endocarditis, especially of the tricuspid valve (TV). Although current guidelines recommend TV repair for native tricuspid valve endocarditis (TVE), the percentage of valve replacements remains very high in numerous studies. The aim of our study is to analyse our experience in the treatment of TVE with a reparative approach.

**METHODS:**

This case series includes all the patients who underwent surgery for acute or healed infective endocarditis on the native TV, at the Cliniques Universitaires Saint-Luc (Bruxelles, Belgium) between February 2001 and December 2020.

**RESULTS:**

Thirty-one patients were included in the study. Twenty-eight (90.3%) underwent TV repair and 3 (9.7%) had a TV replacement with a mitral homograft. The repair group was divided into 2 subgroups, according to whether a patch was used during surgery or not. Hospital mortality was 33.3% (*n* = 1) for the replacement group and 7.1% (*n* = 2) for repair (*P* = 0.25). Overall survival at 10 years was 75.6% [95% confidence interval (CI): 52–89%]. Further, freedom from reoperation on the TV at 10 years was 59.3% (95% CI: 7.6–89%) vs 93.7% (95% CI: 63–99%) (*P* = 0.4) for patch repair and no patch use respectively. Freedom from recurrent endocarditis at 10 years was 87% (95% CI: 51–97%).

**CONCLUSIONS:**

Considering that TVE is more common in young patients, a repair-oriented approach should be considered as the first choice. In the case of extremely damaged valves, the use of pericardial patch is a valid option. If repair is not feasible, the use of a mitral homograft is an additional useful solution to reduce the prosthetic material.

## Graphical Abstract

**Figure:**
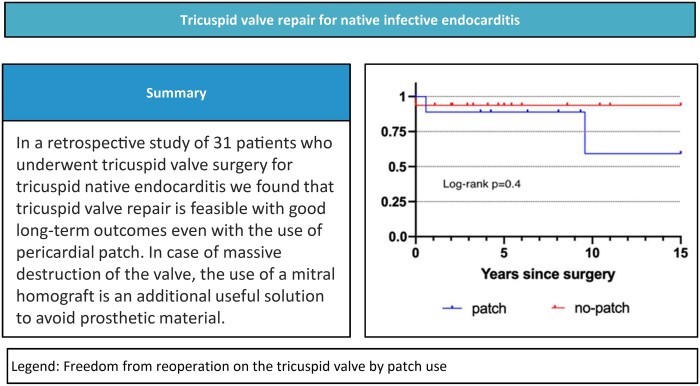

## INTRODUCTION

Despite improvements in diagnosis and management, infective endocarditis (IE) continues to create significant morbidity and mortality due to valvular dysfunctions with congestive heart failures or embolization [1]. As far as tricuspid valve endocarditis (TVE) is concerned, this represents about 5–10% of all IE cases [2, 3]. However, they should not be underestimated, considering the increasing proportion of patients with cardiac implantable electronic devices in the 21st century. In fact, in recent years, this represents an important risk factor for right-side IE, in addition to intravenous drug use, vascular access for dialysis (central venous catheter) and congenital heart diseases [4–6]. It is estimated that the risk of infections after implantation of cardiac pacemaker is 0.5–1% in the first 6–12 months and rises with the increasing complexity of the implanted device [7–9].

Regarding the surgical management, the current American and European Guidelines for the Management of Infective Endocarditis [2, 10] recommend tricuspid valve (TV) repair for native TVE. However, the percentage of valve replacements remains very high in several studies [11].

Through the years, our team has moved towards a more conservative approach to treat active IE.

In this study, we retrospectively review our 20 years’ experience with the surgical treatment of TVE, by analysing the various valve repair techniques, the use of patch and on the long-term outcomes.

## MATERIALS AND METHODS

This case series includes patients admitted to the cardiac surgery unit at the Cliniques Universitaires Saint-Luc (Bruxelles, Belgium) between February 2001 and December 2020, who underwent surgery for acute or healed TVE (patients with inactive endocarditis who nevertheless had lesions on the tricuspid leaflets from previous infection). Diagnosis was based on the revised Duke’s criteria and the European Society of Cardiology (ESC) Guidelines [2, 10].

Patients with a concomitant operation were also included in the final data set, as well as 3 patients with previous TV repair (annuloplasty ring).

Patients were analysed based on the type of surgery (TV repair or replacement with mitral homograft). The repair group was divided into 2 subgroups, according to whether a patch was used during surgery or not.

Preoperative and operative information were extracted from a collected database containing all cardiac procedures performed in our institution.

Demographic and clinical preoperative characteristics are presented in Table 1.

**Table 1:** Patient characteristics.

	Overall (*n* = 31), *n* (%) or mean (SD)^a^	Repair (*n *= 28), *n* (%) or mean (SD)	Replacement (*n* = 3), *n* (%) or mean (SD)	*P*-value	Repair patch (*n* = 10), *n* (%) or mean (SD)	Repair non-patch (*n* = 18), *n* (%) or mean (SD)	*P*-value
Male	18 (58.1)	18 (64.3)	0	0.03	6 (60)	12 (66.7)	0.72
Age (years), mean (SD)	50.9 (17.5)	52.1 (18)	39.4 (5.8)	0.01	49.8 (13.8)	53.4 (20.2)	0.29
History of cardiac surgery	8 (25.8)	8 (28.6)	0	0.28	3 (30)	5 (27.8)	0.9
Previous endocarditis	4 (12.9)	4 (14.3)	0	0.48	1 (10)	3 (16.7)	0.63
Pulmonary embolization	12 (38.7)	10 (35.7)	2 (66.7)	0.29	3 (30)	7 (38.9)	0.64
Cerebral embolization	5 (16.1)	5 (17.9)	0	0.42	2 (20)	3 (16.7)	0.83
Hypertension	11 (35.5)	11 (39.3)	0	0.18	4 (40)	7 (38.9)	0.95
Diabetes mellitus	3 (9.7)	3 (10.7)	0	0.55	2 (20)	1 (5.6)	0.24
Renal impairment (GFR)							
Severe	5 (16.1)	5 (17.9)	0	0.42	2 (20)	3 (16.7)	0.83
Dialysis	5 (16.1)	5 (17.9)	0	0.42	2 (20)	3 (16.7)	0.83
Smoking history	15 (48.4)	13 (46.4)	2 (66.7)	0.51	5 (50)	8 (44.4)	0.78
COPD^b^	3 (9.7)	3 (10.7)	0	0.55	1 (10)	2 (11.1)	0.93
Drugs user	8 (25.8)	6 (21.4)	2 (66.7)	0.09	3 (30)	3 (16.7)	0.41
Pace preoperative	5 (16.1)	5 (17.9)	0	0.42	2 (20)	3 (16.7)	0.83
Urgent	17 (54.8)	16 (57.1)	1 (33.3)	0.43	6 (60)	10 (55.6)	0.82
Emergency	2 (6.5)	2 (7.1)	0	0.63	2 (20)	0	0.04
EuroSCORE II, mean (SD)	7.5 (9)	7.9 (9.4)	4.1 (3.1)	0.09	12 (12.9)	6.14 (7)	0.17

COPD: chronic obstructive pulmonary disease; SD: standard deviation.

a SD: Standard deviation

b COPD: Chronic obstructive pulmonary disease

Postoperative data, clinical and echocardiographic follow-up were collected from patients' medical records.

The primary outcome of the study was survival including in-hospital and late deaths. In-hospital death was defined as any death occurring during the first 30 days after surgery; any other death was considered a late death.

Secondary outcomes included any cardiac reoperation or recurrent endocarditis. Outcomes were reported for the entire cohort and for different subgroups, depending on the type of surgery: repair (with or without patch) and replacement. Operative data are complete, as well as pre- and postoperative characteristics.

Considering the retrospective nature of this study, the Institutional Ethics Review Board of the Cliniques Universitaires Saint Luc, Brussels (2021/10MAI/214), has given its approval, without the need to have the written informed consent.

### Surgical techniques

The interventions were performed by median sternotomy and extracorporeal circulation with aortic cross-clamp. Myocardial protection was achieved by anterograde infusion of warm blood cardioplegia. The TV was exposed via right atriotomy, except for 2 patients with large abscess between mitral-aortic continuity and TV, which were repaired through the aortic access.

Surgical techniques are listed in Table 2.

**Table 2:** Repair surgical technique.

Repair technique.	All (*n* = 28), *n* (%)	Patch group (*n* = 10), *n* (%)	Non-patch group (*n* = 18), *n* (%)	*P*-value
Leaflet sliding	6 (21.4)	2 (20)	4 (22.2)	0.89
Triangular o quadrangular resection	9 (32.1)	3 (30)	6 (33.3)	0.86
Commissure closure	14 (50)	8 (80)	6 (33.3)	0.02
De Vega	5 (17.9)	1 (10)	4 (22.2)	0.42
Artificial neochordae (Goretex CVS)	6 (21.4)	3 (30)	3 (16.7)	0.41
Anulus plication	3 (10.7)	1 (10)	2 (13.3)	0.93
Annuloplasty technique				
Pericardial band	3 (10.7)	2 (20)	1 (5.6)	0.24
Prosthetic ring	8 (28.6)	1 (10)	7 (38.9)	0.1

### Statistical analysis

Continuous data are presented as mean ± standard deviation. Data for categorical variables are reported as frequency and percentage (%). The χ^2^ test and Student’s *t* test have been used to calculate *P*-values for categorical variables and continuous variables respectively. For all analyses, a *P*-value of ≤0.05 was considered significant.

Survival and freedom from reoperation were presented using the Kaplan–Meier curves. Mann–Whitney *U*-test was used to compare the median time of survival between the 2 groups. The long-term outcomes in TV repair with or without patch were compared using the log-rank test. Statistical analyses were performed using GraphPad Prism 9.0.

## RESULTS

Thirty-one patients were included in the study. Seventeen patients (54.8%) were scheduled for urgent surgery, based on septic shock, severe valve regurgitation, mobile or large vegetation with or without systemic embolization and progressive heart failure. All patients received almost 6 weeks of antibiotics treatment. Twenty-seven patients received such a treatment based on positive blood cultures and antibiogram interpretation. *Staphylococcus aureus* was the most common causative organism across all groups. Four patients had negative blood cultures for which empirical therapy was used.

**Figure 1:**
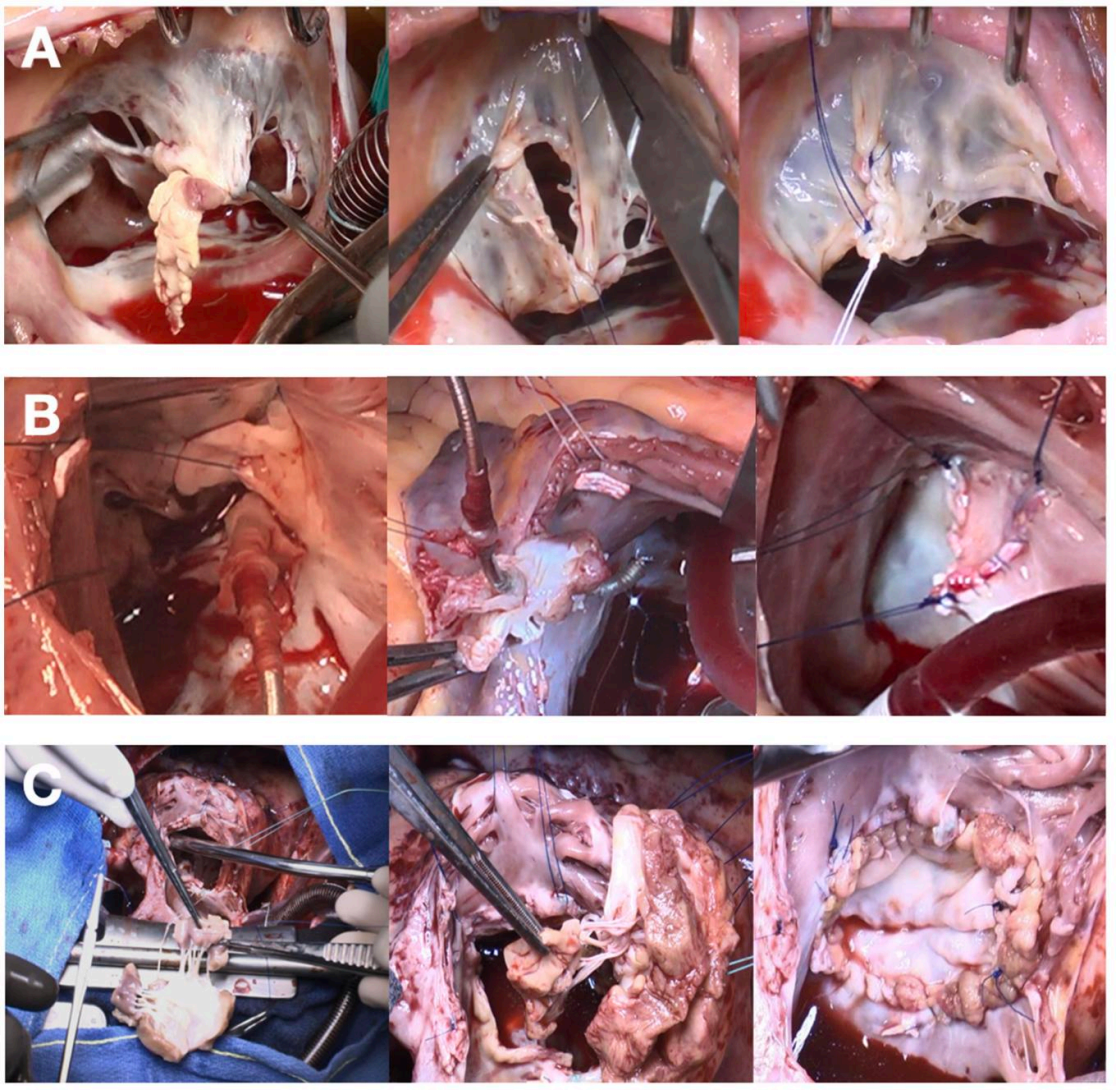
(**A**) Repair without patch; (**B**) repair with patch; and (**C**) replacement of the tricuspid valve with mitral homograft.

Three patients (9.6%) underwent TV replacement with mitral homograft, while 28 patients (90.4%) underwent repair. Patients with a concomitant operation were also included in the final data set. Different types of patches were used according to the surgeon's preference, with a prevalence of fresh autologous pericardium, usually implanted with 2 running sutures (Fig. 1). In 6 cases (21.4%), the repair was also carried out using artificial neochordae. The annuloplasty technique was needed for 11 patients (39.3%). The complexity of the lesions probably explains the longer extracorporeal circulation and cross-clamping time in the patch-repair group (see Supplementary Material, Table S1).

### Early outcomes

No patients died during surgery. At trans-oesophageal echo after weaning from cardiopulmonary bypass (CPB), only 2 patients had moderate residual tricuspid insufficiency, which was accepted due to the complexity of the repair and the risks of a valve replacement with a prosthesis; the others had none- or trivial regurgitation at the end of the procedure.

Postoperative complications are presented in Table 3. Three patients (9.7%) died during the index hospitalization: 2 for multiple-organ failure and 1 for cardiogenic shock. All these patients were operated on in critical conditions and were already on invasive ventilation at the time of surgery. Early (before discharge) TV reoperation was necessary in 1 case in the no-patch repair group for early failure due to suture dehiscence. The patient had a TV replacement with a bio-prosthesis. Perioperative outcomes such as bleeding requiring chest re-exploration, major stroke, pacemaker implantation and hospital lengths of stay were comparable between the groups.

**Table 3:** Complications.

	Overall (*n* = 31)	Repair (*n* = 28)	Replacement (*n* = 3)	*P*-value	Repair patch (*n* = 10)	Repair non-patch (*n* = 18)	*P*-value
Mortality during intervention	0	0	0		0	0	
Postop mortality, *n* (%)	3 (9.7)	2 (7.1)	1 (33.3)	0.14	1 (10)	1 (5.6)	0.66
Chest re-exploration, *n* (%)	3 (9.7)	3 (10.7)	0	0.55	2 (20)	1 (5.6)	0.24
Valve dysfunction, *n* (%)	1 (3.2)	1 (3.6)	0	0.74	1 (10)	0	0.17
Pacemaker implantation, *n* (%)	6 (19.4)	5 (17.9)	1 (33.3)	0.52	3 (30)	2 (11.1)	0.21
Neurological complication, *n* (%)	7 (22.6)	6 (21.4)	1 (33.3)	0.64	3 (30)	3 (16.7)	0.41
*Stroke, n* (%)	2 (6.5)	2 (7.1)	0	0.63	1 (10)	1 (5.6)	0.66
Pneumonia, *n* (%)	5 (16.1)	3 (10.7)	2 (66.7)	0.01	1 (10)	2 (11.1)	0.93
ECMO support, *n* (%)^a^	2 (6.5)	1 (3.6)	1 (33.3)	0.04	1 (10)	0	0.17
Total postoperative stay, days, mean (SD)^b^	21.8 (15.5)	22.7 (15.7)	11 (7)	0.1	28.4 (20.1)	17.8 (11.5)	0.08

ECMO: extracorporeal membrane oxygenation; SD: standard deviation.

a ECMO: extracorporeal membrane oxygenation

b SD: standard deviation

### Late outcomes

**Figure 2:**
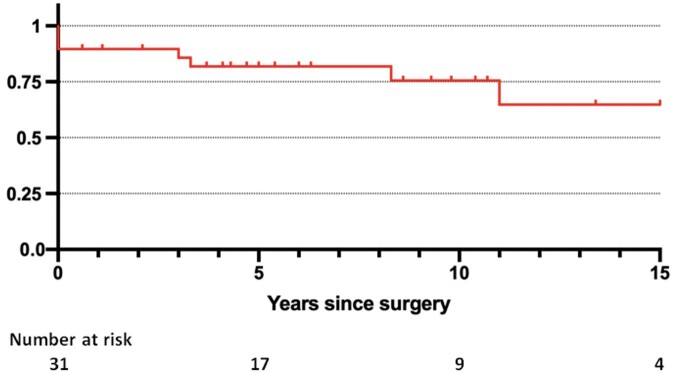
Kaplan–Meier curves showing long-term survival for the entire cohort of patients (*n* = 31).

Two patients were lost during follow-up. During a median follow-up of 95.6 months (interquartile range 58–120), we recorded 5 late deaths (1 death was cardiac related). Overall survival at 5 and 10 years was 81.9% [95% confidence interval (CI): 62–92%] and 75.6% (95% CI: 52–89%), respectively (Fig. 2).

**Figure 3:**
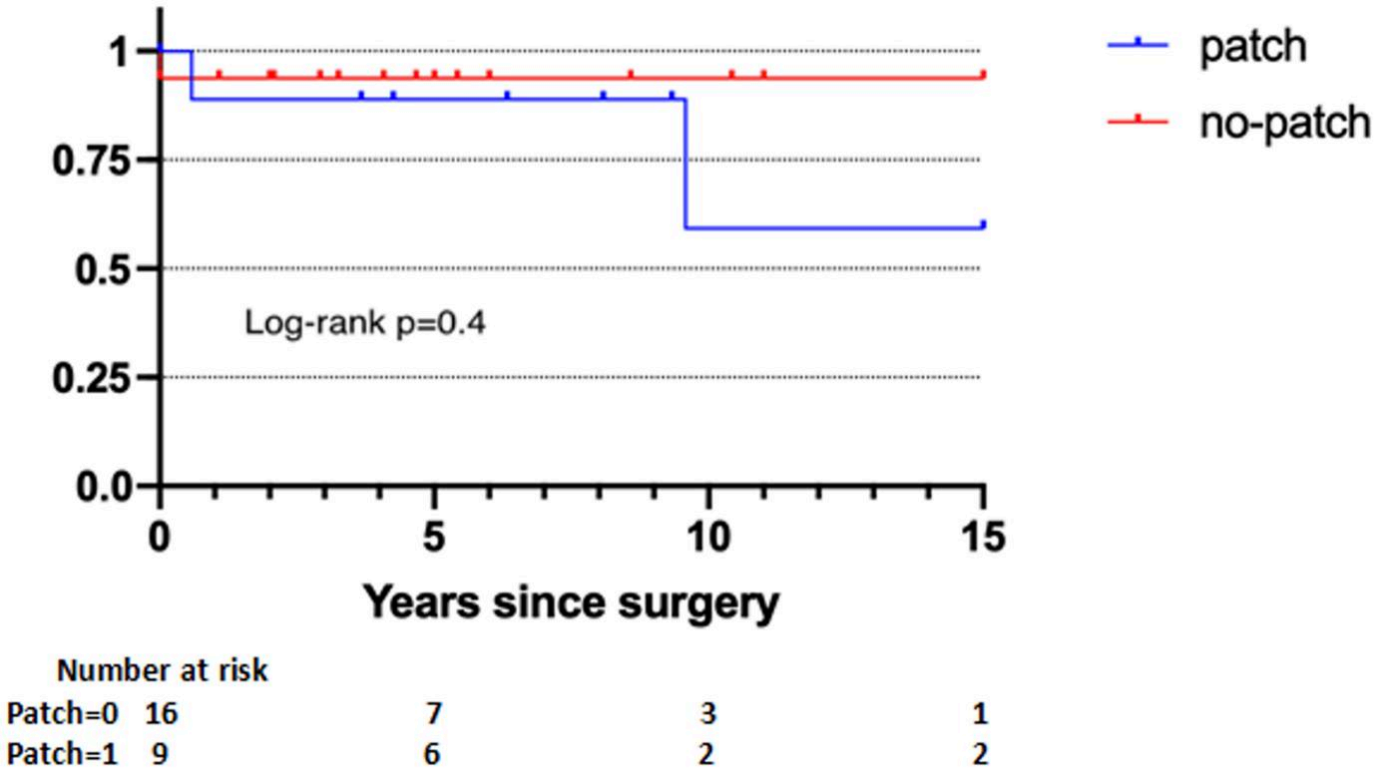
Kaplan–Meier curves showing freedom from reoperation in the repair group with or without patch.

Freedom from all reoperation on the TV at 5 and 10 years was 93% (95% CI: 73–98%) and 81% (95% CI: 41–95%). Analysing the repair group, freedom from reoperation at 10 years was 93.7% (95% CI: 63–99%) and 59.3% (95% CI: 7.6–89%) for no-patch and patch sub-groups, respectively (*P* = 0.4) (Fig. 3). One patient who received TV replacement with a mitral homograft required TV reoperation for homograft stenosis 17 years after surgery.

**Figure 4:**
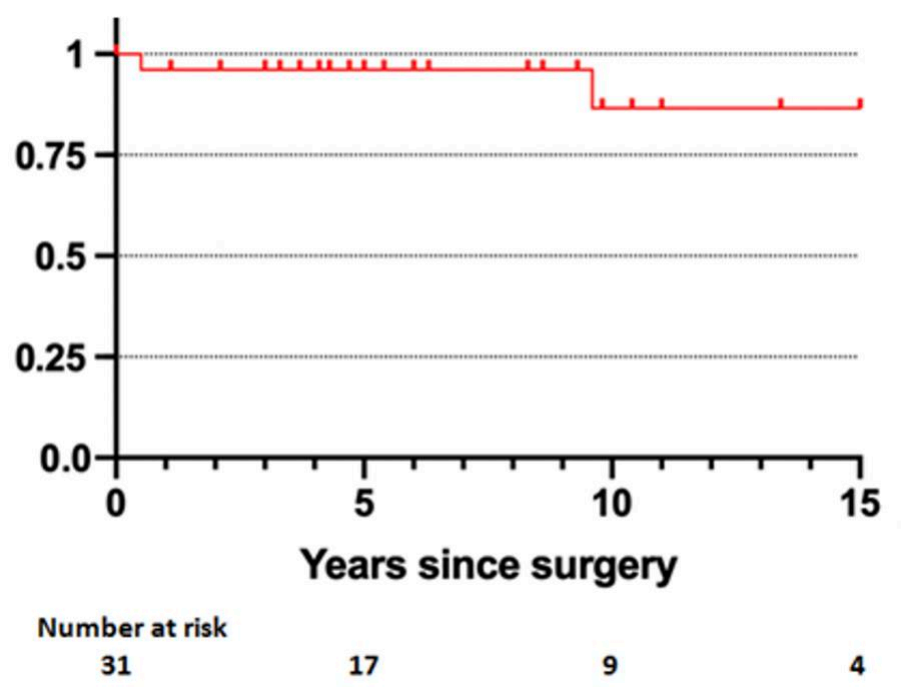
Kaplan–Meier curves showing freedom from recurrent endocarditis.

We did not observe a recurrence of endocarditis in the replacement group. Two patients in the repair group required TV reoperation for recurrent endocarditis during follow-up. One of them received a TV replacement with a mitral homograft due to extensive leaflets destruction 6 months after the first intervention. The other one was re-operated 2 more times: the first one for mitral endocarditis and infection of pacemaker leads but no intervention was required on the TV; unfortunately, the second time he presented a recurrence of IE on the TV that was re-repaired 9 years after the first operation. All patients survived the reoperation. Therefore, freedom from recurrent endocarditis on the TV in the whole cohort was 96% (95% CI: 76–99%) and 87% (95% CI: 51–97%) at 5 and 10 years respectively (Fig. 4).

Regarding freedom from moderate–severe tricuspid regurgitation in the whole cohort, it was 92% (95% CI: 71–98%) at 5 years and 68% (95% CI: 30–88%) at 10 years (see Supplementary Material, Fig. S1).

## DISCUSSION

Infective valve endocarditis still represents a group of diseases with a significant and highly lethal clinical impact [12, 13]. In the literature, there is not a definitive conclusion regarding the best TVE management strategy due to the rarity of the disease precluding large sample sizes, the heterogeneous nature of the pathological process, the use of different techniques among surgeons and the large demographic of patients. These are all factors that affect the comparison of surgical choices [12, 14].

In this 2-decade retrospective study, we reported the surgical treatment and the long-term follow-up of our cohort of patients operated for IE on TV. As for endocarditis of aortic or mitral valve [15, 16] in our institution, we aim to repair the valve to avoid artificial material, lower the risk of recurrent IE and reduce the complications related to both biological and mechanical prostheses [14, 17, 18].

While TV replacement was historically performed more often than repair, in recent years, literature frequently suggests that TV repair is better than replacement, but the percentage of repairs compared to replacements in TVE is still quite low [19].

Our study is one with the highest percentage of repairs compared to replacements in native TVE (over 90% for the whole cohort and 87.5% for isolated TVE) thanks to the use of different repair techniques.

In a recent meta-analysis published in 2018, Yanagawa *et al.* [20] highlighted that valve repair should be the preferred approach because it may offer greater freedom from recurrent endocarditis, re-operations and pacemaker implantations, although without difference in long-term survival after repair or replacement*.* It is noteworthy that these authors considered only isolated TVE and the overall rate of TV repairs was only 59%.

Furthermore, in our analysis, we have shown improved freedom from recurrent endocarditis, reoperation and survival compared with findings in the literature [19, 21–25].

Our freedom from recurrent endocarditis at 10 years (84.1%) was higher than Di Mauro *et al.* [19] (65 ± 5%), although, on the one hand, they analysed isolated tricuspid endocarditis only and, on the other hand, there was a slightly higher percentage of drug users (33% vs 25.8%) in our series. Interestingly, in his study, a TV repair was performed in only 49% of the patients and very rarely (3.2%) a patch was used for repair.

In the study of Dawood *et al.* [21] recurrent TVE was diagnosed in 21% of the replacement group and 0% in the repair group. In our series, we had no recurrent TVE in patients with mitral valve homograft, however we had 2 episodes in the repair group. These patients in our series were both patients at high risk for recurrence indeed: the first one was on chronic dialysis and the second one was intravenous drug user (IVDU) who continued to use drugs after surgery.

Furthermore, analysing the survival rates, our results are better than those of this group of authors (at 5 years: 81.9% vs 76.1%). However, although these authors had a higher proportion of patients with isolated tricuspid operation than us (57% vs 51.6%) they also had a greater percentage of drug users (86% vs 25.8%).

Regarding freedom from reoperation due to reinfections our results at 10 years are similar to those of Musci *et al.* [25] (88.6% vs 87% in our study). These authors interestingly compared survival in right-sided IE and those having combined right and left-sided. They showed a significant difference in survival of patients operated on for right-sided IE compared to patients with combined right- and left-side IE, in favour of the first group of patients. We observed the same result in our cohort (see Supplementary Material, Fig. S2).

Extending the indications of surgery in IVDU remains a controversial issue. Active IVDUs have a higher chance of early recurrent IE after surgery [26]; however, in our study, long-term survival after surgery does not appear to be prohibitive (see Supplementary Material, Fig. S3). The presence of active IVDU does not represent a contra-indication for surgical treatment of TVE, as repetitively reported [8, 20,21, 24]. In a recent analysis of The Society of Thoracic Surgeons national database, Slaughter *et al.* reported the rate of repair at 33% [27]. In our study, the rate of repair in this group of patients was 75%, although lower than non-IVDU (95.4%). Moreover, 2 out of 3 patients in the replacement group were drug users. This can be explained by the fact that in drug users there is a greater destruction of the valve. In fact, in IVDU patients, there is a higher use of the patch compared to non-IVDU (50% vs 31.8%, *P* = 0.4). Regarding 10-year survival, we found a worse outcome, albeit not significant, in non-IVDU compared to IVDU. This surely can be explained by the fact that a statistically significantly higher number of patients in the non-IVDU group underwent also left-sided operations (56.5% vs 12.5%, *P* = 0.03).

In our opinion, the possibility of repairing a TV is based on the extent of the infection and the degree of destruction of the valve. The basic principle for successful surgical treatments of IE, is the radical debridement of the infected tissue in order to prevent further recurrence of the infection. Special attention has to be paid to preserve healthy tissue in order to facilitate the repair process and to ensure effective repair. Pericardial patches are needed in case of large perforation or to reconstruct an extensively destroyed valve. Nonetheless, we found no statistically significant differences in terms of reintervention and recurrent endocarditis between the repair with or without patch.

The high percentage of repair in our series was possible because in cases of tricuspid endocarditis we can tolerate bigger tissue destruction or resection (in case of large vegetation) than what we could accept on the aortic or mitral valve IE. This means that, in the majority of patients, we can remove large parts of the leaflets without jeopardizing the chances for direct repair. Therefore, a complex repair with the use of patch is necessary only in a smaller number of patients. It is indeed of utmost importance to adapt the patch to the lesion and avoid excessive tension on the leaflet that can lead to suture dehiscence. To this end, the use of artificial chordae or reinforcement of the suture on healthy tissue could be helpful. Furthermore, for successful repair, achieving valve coaptation is essential, and the use of De Vega annuloplasty is very beneficial [18, 28]. In patients with significant TV regurgitation, a prosthetic ring would ensure a better result [21]. However, we still prefer not to use prosthetic material in drug users, in order to minimize the risk of reinfections, favouring rather the use of autologous pericardial band for the annuloplasty.

Nonetheless, occasionally the repair can be very challenging due to the extensive lesions that reduce the possibility of preserving native valve tissues and subvalvular apparatus. In these cases, when the TV can by no means be preserved, our choice is to replace the valve with a cryopreserved mitral homograft. Partial or total replacement of the TV with cryopreserved allografts is indeed a valuable option for TVE. Homografts have greater freedom from reinfection compared to xenografts and mechanical valves in aortic and pulmonary positions [29–31]. Although there are not a lot of articles in the literature about the use of mitral homograft in tricuspid position, we believe that a mitral homograft may be as resistant to reinfection as aortic and pulmonary ones. Moreover, as TVE occurs very often in young and middle-aged adults (IVDUs or congenital heart disease patients), mechanical and tissue valves would expose these young patients to the well-known prosthesis-related complications in the long term.

However, in cases where surgeons lack experience for complex valve reconstructions or in institutions where cryopreserved homografts are not available, tricuspid valvectomy may represent another surgical option as a bridge to replacement in patients with drug addiction, after controlling drug dependence [9]. This procedure has been proposed to completely avoid any foreign material, especially in cases of intravenous drug use population or intractable infections [7]. It may be offered as a staged or a palliative procedure, but it is poorly tolerated in patients with moderate–severe pulmonary hypertension, and in our centre, it has never been used.

Nonetheless, complex TV repair and the use of mitral homograft are interventions in which experience plays a fundamental role. As highlighted by Lee *et al.* [23], the percentage of TV repair was higher in hospitals with elevated volume of valve surgery (48% vs 91%). To increase the repair rate and patient’s outcomes, they recommended early referral of patients with TVE to hospitals with a high volume of valve surgery and expert surgeons because in their study the survival benefit of TV repair appeared less prominent in lower volume hospitals when compared with higher volume hospitals.

In conclusion, although many operations were technically challenging, the infected material was removed and the valve function was restored in all patients in our series. The results of this study show that repair of TV in IE, including complex repairs with patch techniques, can achieve good long-term durability, freedom from repairs and re-operations and very low recurrence of endocarditis, even in high-risk patients.

### Limitations

We must acknowledge some limitations of our study, which derive mainly from the limited sample of patients (especially in the replacement group), the large study period and the retrospective nature of this study.

## CONCLUSIONS

Currently, infective valve endocarditis remains a serious life-threatening condition.

A repair-oriented approach, even with the use of pericardial patches, is an effective therapeutic option, with acceptable long-term durability and low recurrence rate of endocarditis. If repair is not feasible, another option to avoid the prosthetic material is the use of a mitral valve homograft.

## Supplementary Material

ivae084_Supplementary_Data

## Data Availability

All the relevant data are within the manuscript and its Supporting Information files. All other information will be shared on reasonable request to the corresponding author.

## Notes

Presented at the HVS Annual Meeting, Eden Roc Miami Beach, USA, 02–05 March 2022.

Presented at the AATS 102nd Annual Meeting, Hynes Convention Center, Boston, MA, USA, 14–17 May 2022.

## ABBREVIATIONS

CI: Confidence interval
IE: Infective endocarditis
IVDUs: Intravenous drug users
TV: Tricuspid valve
TVE: Tricuspid valve endocarditis

## References

[1] Cahill TJ , Prendergast BD. Infective endocarditis. Lancet 2016;387:882–93. 26341945 10.1016/S0140-6736(15)00067-7

[2] Delgado V , Ajmone Marsan N, de Waha S et al; ESC Scientific Document Group. 2023 ESC Guidelines for the management of endocarditis: developed by the task force on the management of endocarditis of the European Society of Cardiology (ESC) Endorsed by the European Association for Cardio-Thoracic Surgery (EACTS) and the European Association of Nuclear Medicine (EANM). Eur Heart J 2023;44(39):3948–4042.37622656

[3] Yong MS , Coffey S, Prendergast BD, Marasco SF, Zimmet AD, McGiffin DC et al Surgical management of tricuspid valve endocarditis in the current era: a review. Int J Cardiol 2016;202:44–8. 26386918 10.1016/j.ijcard.2015.08.211

[4] Mylotte D , Rushani D, Therrien J, Guo L, Liu A, Guo K et al Incidence, predictors, and mortality of infective endocarditis in adults with congenital heart disease without prosthetic valves. Am J Cardiol 2017;120:2278–83. 29103604 10.1016/j.amjcard.2017.08.051

[5] Cahill TJ , Jewell PD, Denne L, Franklin RC, Frigiola A, Orchard E et al Contemporary epidemiology of infective endocarditis in patients with congenital heart disease: a UK prospective study. Am Heart J 2019;215:70–7. 31299559 10.1016/j.ahj.2019.05.014

[6] Athan E , Chu VH, Tattevin P, Selton-Suty C, Jones P, Naber C et al; ICE-PCS Investigators. Clinical characteristics and outcome of infective endocarditis involving implantable cardiac devices. JAMA 2012;307:1727–35. 22535857 10.1001/jama.2012.497

[7] Shmueli H , Thomas F, Flint N, Setia G, Janjic A, Siegel RJ. Right-sided infective endocarditis 2020: challenges and updates in diagnosis and treatment. J Am Heart Assoc 2020;49:e017293. 32700630 10.1161/JAHA.120.017293 PMC7792231

[8] Hussain ST , Witten J, Shrestha NK, Blackstone EH, Pettersson GB. Tricuspid valve endocarditis. Tricuspid valve endocarditis. Ann Cardiothorac Surg 2017;6:255–61. 28706868 10.21037/acs.2017.03.09 PMC5494428

[9] Byrne JG , Rezai K, Sanchez JA, Bernstein RA, Okum E, Leacche M et al Surgical management of endocarditis: the society of thoracic surgeons clinical practice guideline. Ann Thorac Surg 2011;91:2012–9. 21620012 10.1016/j.athoracsur.2011.01.106

[10] Pettersson GB , Coselli JS, Pettersson GB, Coselli JS, Hussain ST, Griffin B et al; Writing Committee. 2016 The American Association for Thoracic Surgery (AATS) consensus guidelines: surgical treatment of infective endocarditis. Executive summary. J Thorac Cardiovasc Surg 2017;153:1241–58.e29. 28365016 10.1016/j.jtcvs.2016.09.093

[11] Gaca JG , Sheng S, Daneshmand MA, O'Brien S, Rankin JS, Brennan JM et al Outcomes for endocarditis surgery in North America: a simplified risk scoring system. J Thorac Cardiovasc Surg 2011;141:98–106.e1-2. 21168017 10.1016/j.jtcvs.2010.09.016

[12] Nappi F , Spadaccio C, Mihos C, Shaikhrezai K, Acar C, Moon MR. The quest for the optimal surgical management of tricuspid valve endocarditis in the current era: a narrative review. Ann Transl Med 2020;8:1628. 33437827 10.21037/atm-20-4685 PMC7791263

[13] Cresti A , Chiavarelli M, Scalese M, Nencioni C, Valentini S, Guerrini F et al Epidemiological and mortality trends in infective endocarditis, a 17-year population-based prospective study. Cardiovasc Diagn Ther 2017;7:27–35. 28164010 10.21037/cdt.2016.08.09 PMC5253443

[14] Gottardi R , Bialy J, Devyatko E, Tschernich H, Czerny M, Wolner E et al Midterm follow-up of tricuspid valve reconstruction due to active infective endocarditis. Ann Thorac Surg 2007;84:1943–8. 18036912 10.1016/j.athoracsur.2007.04.116

[15] Solari S , Tamer S, Aphram G, Mastrobuoni S, Navarra E, Noirhomme P et al Aortic valve repair in endocarditis: scope and results. Indian J Thorac Cardiovasc Surg 2020;36:104–12. 33061191 10.1007/s12055-019-00831-0 PMC7525605

[16] Solari S , De Kerchove L, Tamer S, Aphram G, Baert J, Borsellino S et al Active infective mitral valve endocarditis: is a repair-oriented surgery safe and durable? Eur J Cardiothorac Surg 2019;55:256–62. 30085002 10.1093/ejcts/ezy242

[17] Vaidyanathan K , Agarwal R, Johari R, Cherian KM. Tricuspid valve replacement with a fresh antibiotic preserved tricuspid homograft. Interact CardioVasc Thorac Surg 2010;Jun10:1061–2. 20351018 10.1510/icvts.2010.234757

[18] Akinosoglou K , Apostolakis E, Koutsogiannis N, Leivaditis V, Gogos CA. Right sided infective endocarditis: surgical management. Eur J Cardiothorac Surg 2012;42:470–9. 22427390 10.1093/ejcts/ezs084

[19] Di Mauro M , Foschi M, Dato GMA, Centofanti P, Barili F, Corte AD et al; Italian Group of Research for Outcome in Cardiac Surgery (GIROC). Surgical treatment of isolated tricuspid valve infective endocarditis: 25-years results from a multicenter registry. Int J Cardiol 2019;292:62–7. 31130281 10.1016/j.ijcard.2019.05.020

[20] Yanagawa B , Elbatarny M, Verma S, Hill S, Mazine A, Puskas JD et al Surgical management of tricuspid valve infective endocarditis: a systematic review and meta-analysis. Ann Thorac Surg 2018;106:708–14. 29750928 10.1016/j.athoracsur.2018.04.012

[21] Dawood MY , Cheema FH, Ghoreishi M, Foster NW, Villanueva RM, Salenger R et al Contemporary outcomes of operations for tricuspid valve infective endocarditis. Ann Thorac Surg 2015;99:539–46. 25527426 10.1016/j.athoracsur.2014.08.069

[22] Brescia AA , Watt TMF, Rosenbloom LM, Williams AM, Bolling SF, Romano MA. Patient and surgeon predictors of mitral and tricuspid valve repair for infective endocarditis. Semin Thorac Cardiovasc Surg 2022;34:67–77. S1043-0679(21)00162-3 33865973 10.1053/j.semtcvs.2021.03.017

[23] Lee H-A , Chou A-H, Wu VC-C, Chan Y-S, Cheng Y-T, Chang C-H et al Nationwide cohort study of tricuspid valve repair versus replacement for infective endocarditis. Eur J Cardiothorac Surg 2021;59:878–86. 33156910 10.1093/ejcts/ezaa390

[24] Gaca JG , Sheng S, Daneshmand M, Rankin JS, Williams ML, O'Brien SM et al Current outcomes for tricuspid valve infective endocarditis surgery in North America. Ann Thorac Surg 2013;96:1374–81. 23968767 10.1016/j.athoracsur.2013.05.046

[25] Musci M , Siniawski H, Pasic M, Grauhan O, Weng Y, Meyer R et al Surgical treatment of right-sided active infective endocarditis with or without involvement of the left heart: 20-years single center experience. Eur J Cardiothorac Surg 2007;32:118–25. 17412606 10.1016/j.ejcts.2007.02.034

[26] Shrestha NK , Jue J, Hussain ST, Jerry JM, Pettersson GB, Menon V et al Injection drug use and outcomes after surgical intervention for infective endocarditis. Ann Thorac Surg 2015;100:875–82. 26095108 10.1016/j.athoracsur.2015.03.019

[27] Slaughter MS, , Badhwar V, , Ising M, , Ganzel BL, , Sell-Dottin K, , Jawitz OK et al Optimum surgical treatment for tricuspid valve infective endocarditis: An analysis of the Society of Thoracic Surgeons national database. J Thorac Cardiovasc Surg 2021;161:1227–35.e1. 10.1016/j.jtcvs.2019.10.124 31864695 PMC7310606

[28] Atroshchenko GV , Munoz DE, Jahanyar J, El Khoury G, de Kerchove L. Complex valve reconstruction in isolated tricuspid valve endocarditis. JTCVS Tech 2021;10:293–5. 34977740 10.1016/j.xjtc.2021.05.024 PMC8690277

[29] Gierlinger G , Dolzer-Sames E, Kreuzer M, Mair R, Zierer A, Mair R. Surgical therapy of infective endocarditis following interventional or surgical pulmonary valve replacement. Eur J Cardiothorac Surg 2021;59:1322–8. 33668059 10.1093/ejcts/ezab086

[30] Ostrovsky Y , Spirydonau S, Shchatsinka M, Shket A. Surgical treatment of infective endocarditis with aortic and tricuspid valve involvement using cryopreserved aortic and mitral valve allografts. Interact CardioVasc Thorac Surg 2015 ;20:682–4. 25697982 10.1093/icvts/ivv028

[31] Lorenz V , Carbonez K, de Beco G, Poncelet A. A rare case of infective mediastinitis after melody valve implantation. Congenital Heart Disease 2022;17:187–92.

