# Supplementary material for: Tricuspid valve repair for infective endocarditis
Source: Interdiscip Cardiovasc Thorac Surg. 2024 Apr 30;38(5):ivae084. doi: 10.1093/icvts/ivae084 (PMC11096269; doi:10.1093/icvts/ivae084)
Supplement: ivae084_Supplementary_Data [file ivae084_supplementary_data.zip › Supplementary material revision1.docx]

**Supplementary material**

**Supplementary Table S1**: surgical details

|  | Overall  (n= 31) | Repair  (n= 28) | Replacement  (n=3) | p | Repair  Patch (n=10) | Repair non-patch (n=18) | p |
| --- | --- | --- | --- | --- | --- | --- | --- |
| Total Bypass Time, mean (SD) | 125.3 (60.3) | 123.2(58) | 118.7 (17.1) | 0.32 | 162 (67.6) | 114.1 (60.8) | 0.07 |
| Total cross clamp time, mean (SD) | 93.9 (46.7) | 93.4 (49.1) | 98.3 (11.9) | 0.45 | 113.4 (60.4) | 82.3 (39.1) | 0.11 |
| *Tricuspid alone* | 16 (51.6) | 14 (50) | 2 (66.7) | 0.51 | 6 (60) | 8 (44.4) | 0.28 |
| *Tricuspid + mitral repair* | 5 (16.1) | 5 (17.9) | 0 | 0.42 | 2 (20) | 3 (16.7) | 0.83 |
| *Tricuspid + aortic* | 6 (19.4) | 6 (21.4) | 0 | 0.37 | 2 (20) | 4 (22.2) | 0.89 |
| *Tricuspid + left sided* | 14 (45.2) | 13 (46.4) | 1 (33.3) | 0.66 | 3 (30) | 10 (55.6) | 0.19 |

**Supplementary Figure S1 legend**: Kaplan-Meier curves showing Freedom from moderate- severe tricuspid regurgitation.

**Supplementary Figure S2 legend**: Kaplan-Meier curves showing survival in isolated tricuspid valve surgery and associated with left procedure.

**Supplementary Figure S3 legend**: Kaplan-Meier curves showing survival in IVDU and no IVDU.
