# Supplementary figures and images for: Tricuspid valve repair for infective endocarditis
Source: Interdiscip Cardiovasc Thorac Surg. 2024 Apr 30;38(5):ivae084. doi: 10.1093/icvts/ivae084 (PMC11096269; doi:10.1093/icvts/ivae084)

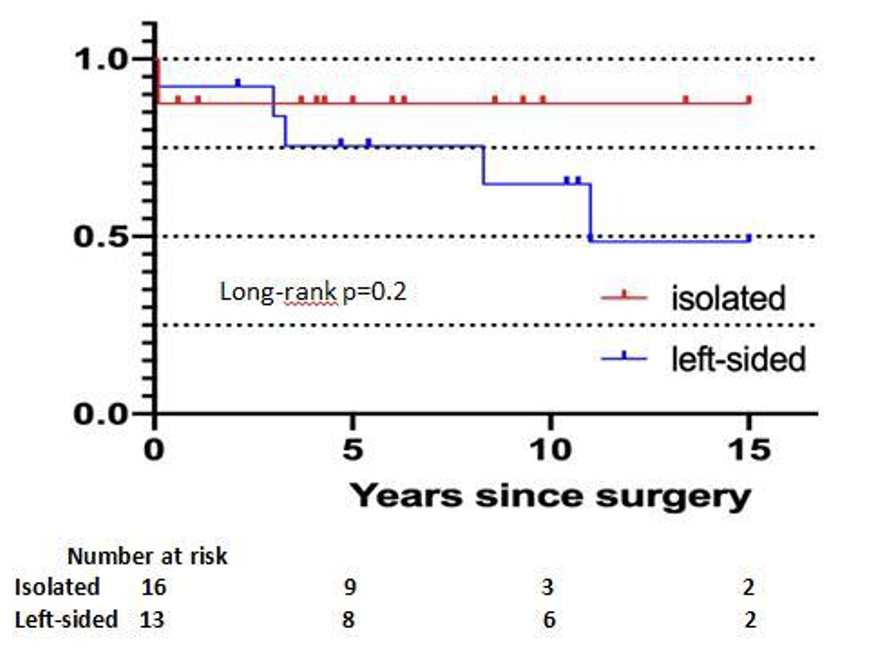

Supplement: ivae084_Supplementary_Data [file ivae084_supplementary_data.zip › Figure S2.png]

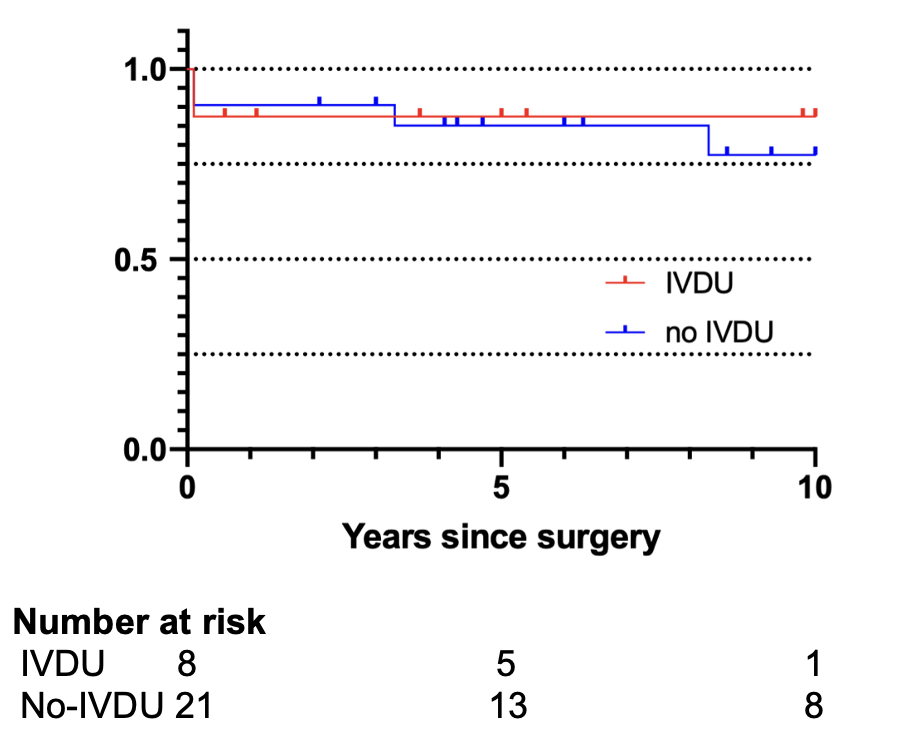

Supplement: ivae084_Supplementary_Data [file ivae084_supplementary_data.zip › Figure S3.png]

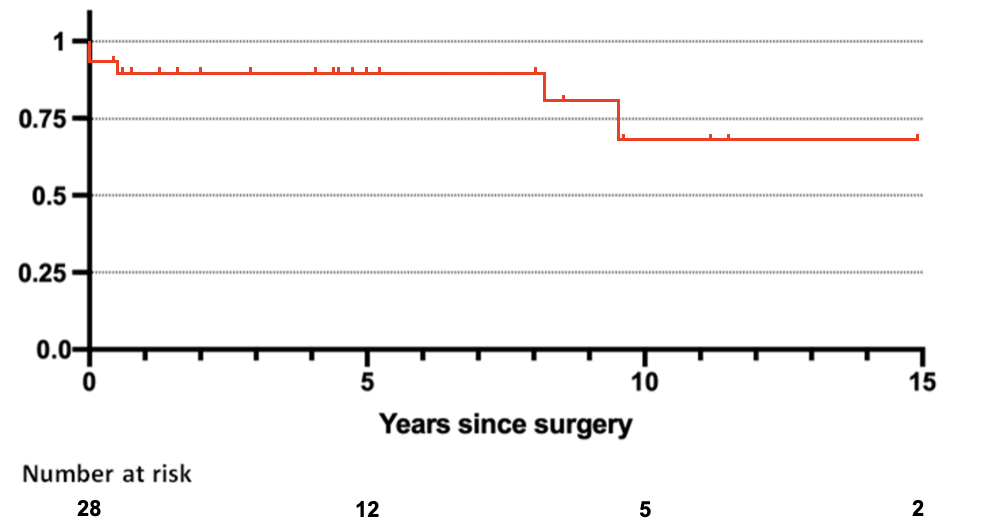

Supplement: ivae084_Supplementary_Data [file ivae084_supplementary_data.zip › Figure S1.png]
